# Agreeable Mode of Taking Castor Oil

**Published:** 1876-04

**Authors:** 


Agreeable Mode of Taking Castor Oil.—Castor oil may be agreeably taken by first rinsing the glass with moderately hot water, and then proceed as follows:

| | |
|---|---|
| Take Hot water, . . . . . | 2 drachms. |
| Castor oil, . . . . . . | 1 ounce. |
| Peppermint water, . . . . | 1 drachm. |

To be put in a tumbler in the order mentioned above. Hardly any taste of the oil remains in the mouth, and it is easily swallowed. The tumbler is also easily cleaned, as little or no oil adheres to it.—*Med. Brief.*

